# Antifungal Activity of Four Medium-Chain Fatty Acids and γ-Undecalactone Against *Candida albicans*

**DOI:** 10.3390/cimb48020150

**Published:** 2026-01-29

**Authors:** Miyako Yoshida, Hiroaki Terada, Saki Hayashi, Tamami Haraguchi, Mayuko Watanabe, Mana Yamashita, Miki Yoshii, Yoshiro Hatanaka, Toshihiro Nagao

**Affiliations:** 1 Department of Clinical Pharmaceutics, Faculty of Pharmaceutical Sciences, Mukogawa Women’s University, 11-68 Koshien 9-Bancho, Nishinomiya City 663-8179, Hyogo, Japan; hayashi_saki_x@mukogawa-u.ac.jp (S.H.); 2016664@mwu.jp (M.W.); 1927817@mwu.jp (M.Y.); 2 FOODLIER Co., Ltd., 3-1 Kamiotsukashinden, Nasushiobara City 329-3134, Tochigi, Japan; terada-hiroaki@foodlier.co.jp; 3 Department of Pharmacy, Meiwa-Hospital, 4-31 Kaminaruomachi, Nishinomiya City 663-8186, Hyogo, Japan; tsuchiko_tamami_x@mukogawa-u.ac.jp; 4 Osaka Research Institute of Industrial Science and Technology, 1-6-50 Morinomiya, Joto-ku, Osaka City 536-8553, Osaka, Japan; yoshii@orist.jp (M.Y.); hatanaka@orist.jp (Y.H.); nagao@orist.jp (T.N.)

**Keywords:** undecanoic acid, undecalactone, antifungal activity, *Candida albicans*, minimum inhibitory concentration

## Abstract

In this paper, the antifungal activity of medium-chain fatty acids with 8 to 11 carbon atoms in their chemical structures, medium-chain fatty acid lactones, and a partially fluorinated medium-chain fatty acid was determined. As the length of the alkyl chain increased in medium-chain fatty acids with 8 to 11 carbon atoms, the MIC and MFC became smaller, with increased antifungal activity (octanoic acid, 225 µg/mL(MIC), 450 µg/mL (MFC); nonanoic acid, 225 µg/mL (MIC), 450 µg/mL (MFC); decanoic acid, 112.5 µg/mL (MIC), 225 µg/mL (MFC); undecanoic acid, 112.5 µg/mL (MIC), 225 µg/mL (MFC)), whereas the antifungal activity of γ-undecalactone, in which the carboxyl group of the medium-chain fatty acid with 11 carbon atoms was converted to a five-membered lactone ring, also had antifungal activity (γ-undecalactone, 112.5 µg/mL (MIC), 225 µg/mL (MFC)). The antifungal activity of the partially fluorinated fatty acid with 11 carbon atoms and δ-undecalactone was not observed and their MICs were not evaluated in this study. The equation derived through multiple regression analysis revealed that the polarizability value was significantly related to the MICs or MFCs of fatty acids containing 8 to 11 carbon atoms and γ-undecalactone (R^2^ = 0.78, *p* < 0.05). *C. albicans* cultured at 37 °C with γ-undecalactone at the MIC formed hyphae or biofilms, which were observed using scanning electron microscopy in this study. Dead *C. albicans* were observed when cultured at 37 °C with γ-undecalactone at the MFC, indicating that in order to demonstrate complete killing, *C. albicans* must be killed at or above the MFC of γ-undecalactone when cultured at 37 °C. γ-undecalactone exhibited no hemolytic activity at the MFC, similar to negative controls. Our results show that γ-undecalactone has an antifungal effect against *C. albicans* over the MFC, without hemodialysis as the observed cytotoxicity.

## 1. Introduction

Organic carboxylic acids with straight or branched, saturated or unsaturated aliphatic chains are called fatty acids [1]. The antimicrobial properties of fatty acids are well established. Fatty acids, which have an antimicrobial effect against pathogens such as bacteria, fungi, and multidrug-resistant bacteria, are found in some algae and plants. Antimicrobially active fatty acids are produced by algae and plants to defend themselves against pathogens [2,3,4]. Such compounds could protect humans against bacterial infections and present an alternative traditional antibacterial agent to use in treatment [5,6,7]. The antibacterial activity of fatty acids against multidrug-resistant bacteria should be a focus point of the Centers for Disease Control and Prevention (CDC). Several reviews describe the antimicrobial activity of fatty acids [3,4,7,8]; however, the detailed mechanisms of action underlying their antibacterial activity remain unclear. For practical use, the antimicrobial activity of fatty acids has been evaluated in combination with other various antibiotics. Fatty acids exert a synergistic effect when combined with penicillin, fluoroquinolones, and aminoglycosides in Gram-positive and Gram-negative bacteria [4,9,10]. Medium-chain fatty acids and their corresponding 1-monoglycerides have a broad spectrum of antimicrobial activity against various viruses and bacteria in vitro [11,12,13,14,15], including pathogens such as herpes simplex virus [16], *Neisseria gonorrhoeae* [17], *Chlamydia trachomatis*, and *Staphylococcus aureus*. The mechanism of action underlying how fatty acids kill bacteria is not clear; however, electron microscopy has indicated that they disrupt cell membranes [18]. Fatty acids are often found in natural products, such as plants and algae, suggesting their nontoxicity to humans at minimum concentrations. Fatty acids can kill various human pathogens and parasites [19].

After being consumed, medium-chain fatty acids are easily converted into energy but not body fat. Medium-chain fatty acids are a key ingredient in human and cow milk, as well as in coconut or palm fruit. They have also been used to supplement postoperative liquid food and premature infant formula for many years. Kabara et al. evaluated the minimum inhibitory concentrations (MICs) of medium-chain fatty acids against *C. albicans* [2]. Medium-chain fatty acids are used as an energy source in the intestinal tract, have the function of preventing the accumulation of body fat, and are considered to be highly functional fatty acids that have antifungal activity against *C. albicans.*

It has been reported that long-chain saturated fatty acids (C18–C22) generally have high antifungal activity [20]. Short-chain fatty acids have been reported to be involved in the metabolism of *C. albicans* [21]. MIC values were not able to be compared because they were affected by differences in experimental conditions. As the antifungal activity of medium-chain fatty acid lactones is still largely unknown, the antifungal activity of medium-chain fatty acids with alkyl chains of similar length to that of medium-chain fatty acid lactones was evaluated by measuring their MICs against *C. albicans* in this study.

Lactones are chemical entities comprising cyclic carboxylic esters. Both natural and synthetic lactones exhibit a broad spectrum of biological activity, including antimicrobial, anti-inflammatory, antihelminthic, and antitumor activities [22,23]. Macrolide antibiotics are composed of a macrocyclic lactone ring connected to cladinose and desosamine, and they belong to the polyketide class of natural compounds. Among macrolide antibiotics, clarithromycin, azithromycin, roxithromycin, and erythromycin tend to be used to treat infections caused by both Gram-positive and Gram-negative bacteria. The antifungal agents commonly used are polyene macrolide antibiotics such as nystatin and amphotericin B [24,25]. Modified γ-lactone has antifungal activity against plant pathogenic fungi [23]. Considering these reports, it is suggested that medium-chain fatty acid lactones may exhibit antifungal activity against pathogenic fungi, including *C. albicans*.

*C. albicans* is an opportunistic microorganism present in the microbiota of skin and mucosa. *C. albicans* can survive outside the body. For example, *C. albicans* survives in soil and water for approximately one month [26,27]. *C. albicans* is dimorphic in that it is able to thrive as either yeast or hyphae. Hyphae serve as a pivotal virulence factor essential for biofilm formation [28]. As drug-resistant *C. albicans* and *C. auris* (another drug-resistant fungi) are now widespread, novel therapeutics that modulate host immunity and target virulence traits are being explored to combat antifungal resistance [29,30]. *C. albicans* has numerous virulence factors, including lipases, phospholipases, adhesins, and aspartyl proteases, as well as the ability to form biofilms [31]. Especially, the destruction or inhibition of *C. albicans* biofilm formation on indwelling medical devices and host tissues represents an important issue when conducting clinical treatment. Many approaches have been devised for controlling *C. albicans* biofilms [32,33].

Inhibiting *C. albicans* is important for medical treatments. Whether medium-chain fatty acid lactones have antifungal activity against *C. albicans* is unclear; however, it is possible that medium-chain fatty acid lactones, with a similar alkyl chain structure as that of medium-chain fatty acids, shows antifungal activity against *C. albicans* given that medium-chain fatty acids with the same number of carbons in the structure show antimycotic activity against *C. albicans* [34]. In this study, it was investigated whether medium-chain fatty acids with 8 to 11 carbon atoms, undecalactones, and a partially fluorinated fatty acid with 11 carbon atoms exhibit antifungal activity against *C. albicans*, and by analyzing which physicochemical factors contribute to the antifungal activity, it was considered which functional groups in the compounds contribute to the mechanism underlying their antifungal activity.

## 2. Materials and Methods

### 2.1. Materials

Medium-chain fatty acids (octanoic acid, nonanoic acid, decanoic acid, and undecanoic acid) (MCFA1 to MCFA4), γ-undecalactone (γ-UDL), and δ-undecalactone (δ-UDL) were purchased from Tokyo Chemical Industry Co., Ltd. (Tokyo, Japan). 4,4,5,5,6,6,7,7,8,8,9,9,10,10,11,11,11-Heptadecafluoroundecanoic acid (HFDEC) was purchased from Merck KGaA (Darmstadt, Germany). All chemicals were dissolved in DMSO and were set at 18 mg/mL. The microbial strain used here was *C. albicans* JCM1542^T^, which was purchased from the Japan Collection of Microorganisms, RIKEN BioResource Research Center (Ibaragi, Japan). *C. albicans* JCM1542^T^ was pre-cultivated in Yeast Extract-Malt Extract broth (YM broth) at pH 6.2 and 37 °C for 48 h before being used in the experiment.

### 2.2. Determination of Minimum Inhibitory Concentration (MIC)

MIC was determined as previously described [35,36,37,38], with minor modifications. The *C. albicans* strain was diluted to 4 × 10^4^ CFU/mL in Yeast Extract-Malt Extract broth (YM broth) at pH 6.2. Next, 15 µL of sample solution dissolved in DMSO and 285 µL of bacterial solution were added to the leftmost well of the plate, bringing the total volume to 300 µL. A volume of 150 µL of bacterial solution was added to each well in advance, and 150 µL was taken from the left well and transferred to the right well. After mixing the 300 µL in the well, another 150 µL was transferred to the right well. By repeating this procedure, two-step dilution of the sample was performed. The plates were incubated at 37 °C for 48 h after dispersing 150 µL of the culture into a 96-well microtiter plate well. The MIC value was set as the minimum concentration of the antifungal substance necessary to prevent fungal growth after 48 h of incubation at 37 °C. The minimum sample concentration in the well where no fungal growth was visible was determined as the MIC.

### 2.3. Determination of Minimum Fungal Concentration (MFC)

A volume of 2 µL was removed from the wells in the microtiter plates where no growth was observed after 48 h of incubation at 37 °C and then inoculated onto the surface of YM broth agar plates. The plates were incubated for 48 h at 37 °C, with the MFC taken as the lowest concentration of the substance at which no colonies formed under these conditions. Each analysis was performed in triplicate.

### 2.4. Physical Property–Antifungal Activity Correlation at MIC Through Multiple Regression Analysis

The physicochemical parameters of MCFA1 to MCFA4, γ-UDL, δ-UDL, and HFDEC were derived from MarvinSketch (VERSION 20.4, Certara, Radnor, PA, USA). The 11 physicochemical parameters used here were as follows: molecular weight (MW), pKa, ion form fraction (pH 6.2), molecular form fraction (pH 6.2), log *P*, log *D* (pH 6.0), log *S* (pH 6.0), and polarizability (isotopic, tensor component xx, tensor component yy, and tensor component zz). Multiple regression analysis was performed to evaluate the correlation between the MICs of MCFA1 to MCFA4 and γ-UDL and their physicochemical properties, with physicochemical properties as explanatory variables and MICs as target variables.

### 2.5. Observation of Dead Bacteria Using Propidium Iodide (PI) Staining

*C. albicans* cells were cultured in YM broth (4 × 10^4^ CFU/mL). Next, the sample solution was added to the *C. albicans* culture medium in six wells of a 24-well plate to prepare 2 mL of MFC solution for each sample (δ-UDL and HFDEC were set at 450 µg/mL, which was the highest MIC of MCFA) per well. After 48 h incubation at 37 °C, the MFC solution for each sample of six wells was collected and centrifuged (2000 *g*, 25 °C, 10 min). The supernatant was removed and the *C. albicans* cells were concentrated 10 times. Furthermore, the collected sample solution was centrifuged (2000 *g*, 25 °C, 10 min). The supernatant was removed and the *C. albicans* cells were concentrated 100 times. Finally, 7.5 µL of 2 µg/mL PI solution was added to 15 µL of YM broth with *C. albicans*, incubated at 37 °C for 30 min, and observed under a fluorescence microscope.

### 2.6. Observation of Morphological Changes on Fungal Surfaces Using Scanning Electron Microscope (SEM)

Morphological observation of *C. albicans* via SEM (JSM-7800, JEOL Ltd., Tokyo, Japan) was performed as previously described [35,36], with minor modifications. Briefly, *C. albicans* cells were cultured in YM broth (4 × 10^4^ CFU/mL). Next, the sample solution was added to the *C. albicans* culture medium in four wells of a 24-well plate to prepare an MIC solution for each sample (δ-UDL and HFDEC were set at 225 µg/mL, which was the highest MIC of MCFA), and the total volume was adjusted to 2 mL per well. After incubation, the *C. albicans* cells in four well were concentrated 100 times and fixed using 2.5% glutaraldehyde at 4 °C overnight. After washing with phosphate-buffered saline twice, the *C. albicans* cells were fixed using 1% OsO_4_ for 2 h. The cells were dehydrated using a graded ethanol solution series, placed in t-butanol and freeze-dried, and platinum was coated on the surface of the cells. The platinum-coated cells were observed using SEM.

### 2.7. Hemolytic Analysis

The hemolytic activity was evaluated using sheep red blood cells with a modified previously reported method [39]. First, 3 mL of sterile defibrinated whole sheep blood was mixed with 3 mL of saline and stirred, then centrifuged (23,800 *g*, 20 min), and the supernatant was removed. This procedure was repeated five times, and saline was added to the residue to make the total volume 500 µL. As a red blood cell solution, the blood cell count was approximately 200 × 10^3^/mL. As a sample solution to prepare an MFC solution for each sample (δ-UDL and HFDEC were set at 450 µg/mL, which was the highest MFC of MCFA), the total volume was adjusted to 200 µL using PBS. As a positive control, 200 µL of 10% Triton X in PBS was prepared. Next, 50 µL of red blood cell solution was added to 200 µL of sample solution and incubated at 37 °C for 1 h. Then, the mixture was centrifuged at 15,000 *g* for 10 min. The absorbance of 200 µL of the supernatant was measured at a wavelength of 450 nm.

### 2.8. Statistic Analysis

BellCurve from Excel (Ver.3.20, Social Survey Research Information Co., Ltd., Tokyo, Japan) was used for statistical analysis using multiple regression analysis. A 5% level of probability was considered significant. Pearson’s correlation test was performed using Excel.

## 3. Results

### 3.1. MICs and MFCs of MCFAs, UDLs, and HFDEC

**Figure 1:**
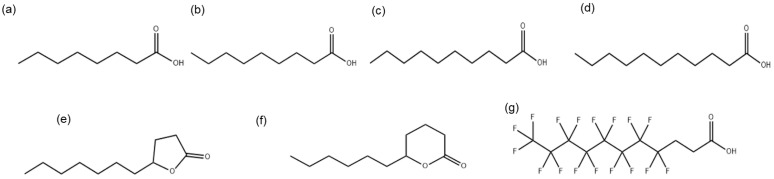
Chemical structures of medium-chain fatty acids: (**a**) octanoic acid (MCFA1), (**b**) nonanoic acid (MCFA2), (**c**) decanoic acid (MCFA3), (**d**) undecanoic acid (MCFA4), lactones (**e**) γ-undecalactone (γ-UDL), (**f**) σ-undecalactone (δ-UDL), and (**g**) partially fluorinated fatty acid 4,4,5,5,6,6,7,7,8,8,9,9,10,10,11,11,11-heptadecafluoroundecanoic acid (HFDEC).

The MICs were determined for compounds (a) to (g) shown in Figure 1.

The chemical structures of medium-chain fatty acids MCFA1, MCFA2, MCFA3, MCFA4, γ-UDL, δ-UDL and HFDEC are presented in Figure 1. Hereafter, the compounds in the text are shown in the numerical order of Figure 1.

The MIC values are shown in Table 1. The MIC values of MCFA1 to MCFA4 decreased with extension of the alkyl group in their structures. Accordingly, the length of the alkyl group in MCFA1 to MCFA4 was suggested to enhance their antifungal activity. Because the MIC of γ-UDL showed a smaller value, nearly the same as MCFA3 and MCFA4, it had the more potent antifungal activity among MCFA1 to MCFA4. The MICs of δ-UDL and HFDEC were determined to be over 900 µg/mL and showed little antifungal activity. The long length of the alkyl group was suggested to enhance the potency of antifungal activity in MCFA1 to MCFA4. The MICs of δ-UDL and HFDEC were not obtained in this study.

### 3.2. Physicochemial Characteristics of MCFAs, UDLs, and HFDEC

The following physicochemical properties of the seven compounds, MCFA1 to MCFA4, γ-UDL, δ-UDL, and HFDEC, except for the molecular weight (MW), were obtained using MarvinSketch: pKa, ion form fraction (pH 6.2), molecular form fraction (pH 6.2), log *P*, log *D* (pH 6.0), log *S* (pH 6.0), and polarizability (isotopic, tensor component xx, tensor component yy, and tensor component zz). These are summarized in Table 2. To investigate the contribution of the physicochemical properties (explanatory variables) to the obtained MICs of the five chemicals against *C. albicans* (target variable), multiple regression analysis was performed, and partial regression coefficients were obtained. The following multiple regression formulas were obtained from multiple regression analysis:

Predicted MIC (µg/mL) = −21.8 Polarizability + 606(1)

Predicted MFC (µg/mL) = −43.7 Polarizability + 1214(2)

It is known that polarizability affects van der Waals forces, which are one of the intermolecular attractive forces, and it was found that van der Waals forces may contribute to MIC and MFC. Polarizability was found to have a significant multiple correlation coefficient (R^2^ = 0.78, *p* < 0.05). The multiple correlation coefficient R^2^ is a number that indicates the strength of the correlation between the predicted values obtained by multiple regression analysis and the actual observed values. R^2^ takes values between 0 and 1, and a value closer to 1 indicates a stronger correlation (higher predictive accuracy). In this study, the value of R^2^ was 0.78, close to 1, suggesting high predictive accuracy.

**Figure 2:**
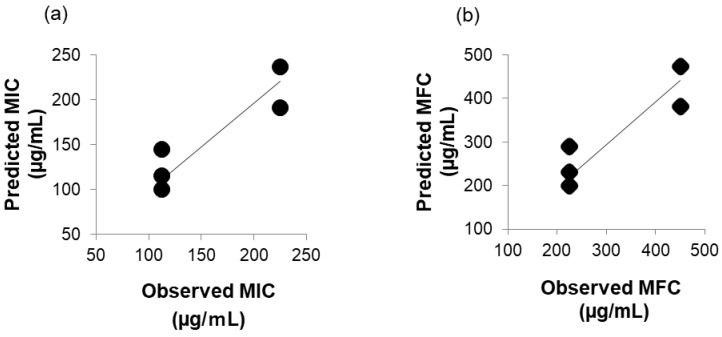
(**a**) Correlation between predicted MIC (µg/mL) and observed MIC (µg/mL). (**b**) Correlation between predicted MFC (µg/mL) and observed MFC (µg/mL).

In Figure 2a, the correlation between predicted MIC (µg/mL) and observed MIC (µg/mL) is shown, and in Figure 2b, the correlation between predicted MFC (µg/mL) and observed MFC (µg/mL) is shown. The Pearson’s correlation coefficient was 0.91, respectively. A strong positive correlation between predicted MIC (µg/mL) or MFC (µg/mL) predicted using the multiple regression formula and observed MIC (µg/mL) or MFC (µg/mL) observed in vitro was shown. It was thought to be caused by intermolecular interactions with the membrane surface of *C. albicans*, but it was not clear which membrane component was interacting. This is a topic for future investigation.

### 3.3. Observation of Dead Fungi Using PI Staining of MCFAs, UDLs, and HFDEC

**Figure 3:**
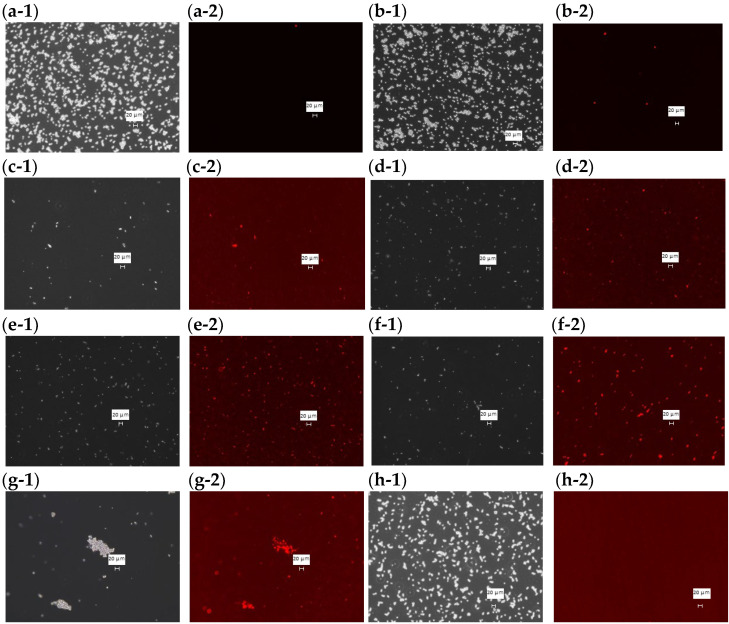
PI staining of dead cells at the MFC of each sample. (**a-1**) Phase contrast microscope image with DMSO (2.5%), (**a-2**) fluorescence microscopy image with DMSO (2.5%), (**b-1**) phase contrast microscope image with HFDEC, (**b-2**) fluorescence microscopy image with HFDEC, (**c-1**) phase contrast microscope image with MCFA1, (**c-2**) fluorescence microscopy image with MCFA1, (**d-1**) phase contrast microscope image with MCFA2, (**d-2**) fluorescence microscopy image with MCFA2, (**e-1**) phase contrast microscope image with MCFA3, (**e-2**) fluorescence microscopy image with MCFA3, (**f-1**) phase contrast microscope image with MCFA4, (**f-2**) fluorescence microscopy image with MCFA4, (**g-1**) phase contrast microscope image with γ-UDL, (**g-2**) fluorescence microscopy image with γ-UDL. (**h-1**) phase contrast microscope image with δ-UDL, (**h-2**) fluorescence microscopy image with δ-UDL. The scale bar is indicated in the figure as 20 µm.

Phase contrast and fluorescence microscope images were taken of PI-stained *C. albicans* with each sample added at the MFC and incubated at 37 °C for 48 h (Figure 3). In the fluorescence images of DMSO (Figure 3a-2), HFDEC (Figure 3b-2), and δ-UDL(Figure 3h-2), little cell death was observed since most fungal cells were not stained. In the fluorescence images of MCFA1–4 (Figure 3c-2–f-2) and γ-UDL (Figure 3g-2), many fungal cells were stained red, indicating cell death.

### 3.4. Scanning Electron Microscopy (SEM) of MCFAs, UDLs, and HFDEC

**Figure 4:**
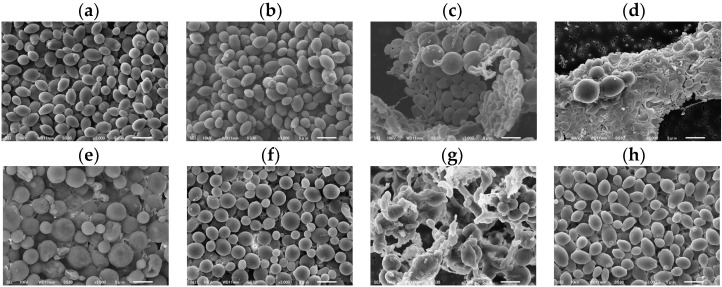
SEM images of *C. albicans* after incubation for 48 h with sample at MIC. (**a**) DMSO (1.25%), (**b**) HFDEC, (**c**) MCFA1, (**d**) MCFA2, (**e**) MCFA3, (**f**) MCFA4, (**g**) γ-UDL, and (**h**) δ-UDL. The scale bar is indicated at the bottom of the figure as 5 µm.

*C. albicans* morphological changes were observed after 37 °C incubation with the sample at the MIC for 48 h using SEM (Figure 4). There were no morphological changes at the MIC of DMSO (1.25%) (Figure 4a), HFDEC (Figure 4b), and δ-UDL (Figure 4d). Morphological changes such as round ball-like spheres from the original oval shapes were observed at the MIC of MCFA1–4 (Figure 4c–f). Regarding the morphology of *C. albicans* at the MIC of γ-UDL (Figure 4g), in addition to hyphae, biofilms were also observed.

### 3.5. Hemolytic Activity of MCFAs, UDLs, and HFDEC

**Figure 5:**
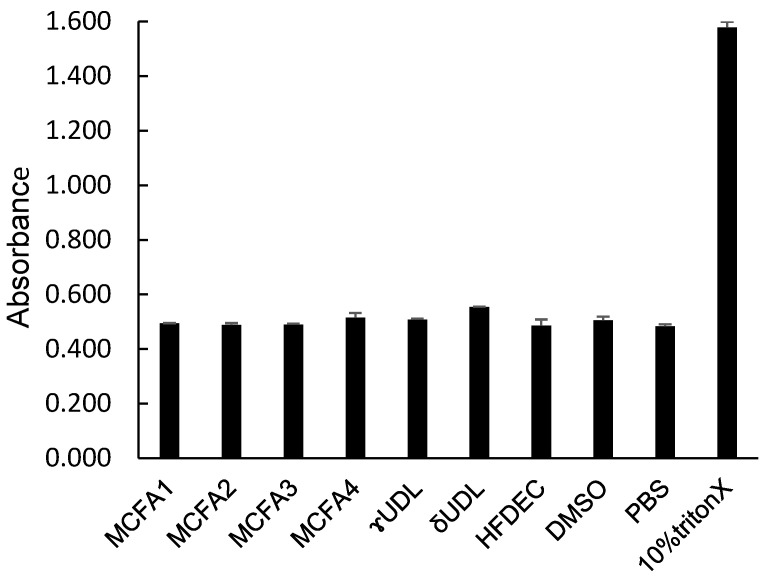
Absorbance of sample solution after hemolytic reaction. *n* = 3, mean ± SD.

While the destructive effects of MCFAs and UDLs against the unicellular microorganism *C. albicans* have been clarified, their hemolytic effect was investigated to examine their destructive effects (cytotoxicity) on the cells of *C. albicans*. The hemolytic activity was measured as an index of cytotoxicity. The results are shown in Figure 5. Only the positive control, 10% Triton X, exhibited hemolytic activity, resulting in a high absorbance value. MCFAs, UDLs, and HFDEC all exhibited absorbance values that were similar to those of the negative controls, PBS and DMSO. These results demonstrated that these compounds did not exhibit hemolytic activity at the MFC.

## 4. Discussion

In this report, it was shown that γ-UDL exhibited antifungal activity against *C. albicans* over the MFC. Hyphae and biofilms were also observed at the MIC of γ-UDL, although it inhibited the cell proliferation of *C. albicans.* It was shown that for γ-UDL to reliably kill *C. albicans*, a concentration higher than the MFC, rather than the MIC, which inhibits cell growth, is required.

It was shown that the longer the alkyl chain, the lower the MIC, comparing MCFAs from C8 to C11. According to some reports, when comparing the MICs of MCFAs from C8 to C11, the longer the alkyl chain, the lower the MIC [2,13,15], which supports our results. The alkyl chain of γ-UDL was longer than that of δ-undecalactone, and the MIC was lower, suggesting that the length of the alkyl chain may also affect the MIC of lactones. However, the lactones used as food additives and quasi-drugs were racemic mixtures. How the chirality of γ-UDL could influence its antifungal properties is unclear.

A strong regression is considered to exist when the coefficient of determination of multiple regression analysis is 0.5 or higher. The obtained coefficient of determination was 0.78, suggesting that it was possible to predict the MICs of compounds (a) to (f) using the polarizability value. A strong correlation is considered to exist when the Pearson’s correlation coefficient is between 0.7 and 1.0. The Pearson’s correlation coefficient between the observed and predicted MICs was 0.91, suggesting that the multiple regression equation for predicting MICs derived through multiple regression analysis could predict the observed MICs of MCFAs and γ-UDL.

In the fluorescence images of *C. albicans* after PI staining at the MFC of MCFA1 to MCFA4 and γ-UDL, many fungal cells were stained red, indicating cell death. However, the mechanism is unclear. From the results in this study, MCFA1 to MCFA4 and γ-UDL may attack the cell body surface of *C. albicans*.

The surface membrane composition of *C. albicans* is closely linked to its infection-related functions and drug resistance. Therefore, a detailed understanding of its membrane composition is essential for developing antifungal drugs and improving infection control strategies. The surface of *C. albicans* contains several important characteristic components in the cell wall and membrane. The cell wall of *Candida* is mainly composed of mannan, β-glucan, and chitin. Mannan is located in the outer layer of the cell wall, where it plays an important role in fungal recognition and immune responses, as well as in pathogenesis. β-glucan is abundant in the inner layer of the cell wall, where it provides structural strength. β-glucan is also recognized by specific immune cell receptors (e.g., Dekton-1) and triggers an immune response. Chitin plays a role in increasing the stability and resistance of the cell wall and is present in the lower layer of β-glucan. The cell membrane of *Candida* is also composed of phospholipids and ergosterol (a sterol unique to fungi). Ergosterol regulates membrane fluidity and permeability and is a target for antifungal drugs (e.g., azoles) [40]. The membrane composition of *C. albicans*, which has a multilayered structure, supports its high environmental adaptability and pathogenicity, making treatment challenging. Considering the composition of lactones and the cell surface of *C. albicans*, the possibility of binding reactions such as hydrophobic bonds and hydrogen bonds was considered, so molecular weight, hydrophobicity, hydrophilicity, and polarizability were selected as physicochemical parameters. A strong positive correlation between predicted MFC and observed MFC was shown (Pearson’s correlation coefficient: 0.91). The predicted formula for MFC derived from regression analysis demonstrated that polarizability was important for the antifungal activity represented by the MFC. In order for γ-UDL to reliably kill *C. albicans*, a concentration above the MFC, rather than the MIC, which inhibits cell growth, was required. This suggested that the addition of γ-UDL at a concentration above the predicted MFC is necessary to reliably kill fungi. The SEM results suggested that *C. albicans* cultured at 37 °C with γ-UDL at the MIC formed hyphae or biofilms. *C. albicans* must be killed at or above the MFC of γ-UDL when cultured at 37 °C; otherwise, even at an MIC lower than the MFC of lactones, *C. albicans* may be at risk of forming hyphae or biofilms.

In the case that the hydrogen of the alkyl group was partially replaced with fluorine, the antifungal activity disappeared, suggesting that the presence of the alkyl group plays an important role in the hydrophobic interactions that induce inward indentations. Although not listed in Table 1, 4,4,5,5,6,6,7,7,8,8,9,9,9-tridecafluorononanoic acid also had no antifungal activity. Results suggested that partial fluorination of the alkyl chains of the fatty acids partially increased their polarity, resulting in a decrease in antifungal activity with the lipophilic membrane components of the fungus, allowing it to adsorb to the membrane surface, resulting in inward indentations. These results suggested that the alkyl chains of fatty acids have hydrophobic interactions with the substances present on the surface of *C. albicans*. Furthermore, it was predicted that the decrease in antifungal activity due to partial fluorination of the alkyl chains would also apply to undecalactones. The mechanism by which undecalactones exhibit antifungal activity involves hydrophobic interactions between the alkyl chains of the compounds themselves and the substances on the surface of *C. albicans*, as supported by multiple regression analysis showing that high polarizability contributes to low MICs (high antifungal activity).

*C. albicans* grown at room temperature, such as 27 °C, is more virulent than at mice body temperature [41]. MIC measurements were performed at 37 °C in this study, the temperature at which *C. albicans* grows easily and is highly infectious. The MIC, an index of bacteriostatic activity, is used based on the idea that if the growth of pathogenic microorganisms can be prevented in the case of infection in patients with normal immune function, they will be killed by the immune system and expelled from the body. Although not shown as data, when *C. albicans* was cultured at 37 °C, which is close to the in vivo environment, it grew faster than when it was cultured at 27 °C, and the antifungal effects of MCFA and γ-UDL were weakened. *C. albicans* cultured at 37 °C with γ-UDL at the MIC formed hyphae or biofilms. Dead *C. albicans* were observed when cultured at 37 °C with γ-UDL at the MFC, indicating that in order to demonstrate complete killing, *C. albicans* must be killed at or above the MFC of γ-UDL when cultured at 37 °C.

γ-UDL is used as a food additive and is very safe and not as expensive as antifungal substances. MCFAs, UDLs, and HFDEC all exhibited no hemolytic activity, similar to the negative controls PBS and DMSO. Only the positive control, 10% Triton X, exhibited hemolytic activity. These results showed that these compounds do not exhibit hemolytic activity at the MFC.

It has been reported that MCFA4 may inhibit ergosterol synthase in the mycelium of *C. albicans*. Azole antifungal drugs are also known to inhibit ergosterol synthase. When MCFA4 and the azole antifungal drug, miconazole, were added to a culture medium of *C. albicans* and the morphology of the fungus was observed, the fungus was confirmed to be crushed [35].

The mechanism of the antifungal action of γ-UDL against *C. albicans* is unclear. Phospholipids such as phosphatidylcholine and phosphatidylethanolamine, as well as ergosterol, are known to be present in the cell membrane of *C. albicans*. Ergosterol is the target molecule of the polyene antifungal drug amphotericin B [42]. Since lactones had higher antifungal activity than fatty acids with the same number of carbon atoms, it is thought that the alkyl chain portion of lactones may contribute to their antifungal activity, and therefore it is possible that they form hydrophobic bonds with the alkyl chains of phospholipids or ergosterol present in the cell membrane. Ergosterol, present in fungal cell membranes, binds to fatty acids through hydrophobic interactions and maintains the structure and function of the cell membrane. This interaction is important for regulating membrane fluidity and permeability [43].

*C*. *albicans* resistance to azole and polyene antifungal agents has emerged. Mechanisms of resistance to azole antifungal agents include enhanced drug efflux from cells (hyperfunction of the efflux pump, CDR) and increased or structural changes in CYP51, which is involved in ergosterol synthesis. One of the mechanisms of resistance to polyene antifungal agents is the generation of ergosterol with low affinity for polyenes due to changes in the ergosterol synthesis pathway.

Our results in this paper show the possibility that γ-UDL can kill *C*. *albicans*, which causes opportunistic infections, at a concentration that does not cause hemolysis.

## 5. Conclusions

In this paper, the antifungal activity of medium-chain fatty acids with 8 to 11 carbon atoms in their chemical structures, medium-chain fatty acid lactones, and a partially fluorinated medium-chain fatty acid was determined. As the length of the alkyl chain increased in medium-chain fatty acids with 8 to 11 carbon atoms (MCFAs), the MIC and MFC became smaller, with increased antifungal activity, whereas the antifungal activity of γ-UDL, in which the carboxyl group of the medium-chain fatty acid with 11 carbon atoms was converted to a five-membered lactone ring, also had antifungal activity. The antifungal activity of the partially fluorinated fatty acid with 11 carbon atoms and δ-UDL was not observed in this study. The equation derived through multiple regression analysis revealed that the polarizability value was significantly related to the MICs or MFCs of MCFA and γ-UDL (R^2^ = 0.78, *p* < 0.05). *C. albicans* cultured at 37 °C with γ-UDL at the MIC formed hyphae or biofilms, as observed via scanning electron microscopy in this study. Dead *C. albicans* were observed when cultured at 37 °C with γ-UDL at the MFC, indicating that in order to demonstrate complete killing, *C. albicans* must be killed at or above the MFC of γ-UDL when cultured at 37 °C. No hemolytic activity was observed at the MFC of γ-UDL, similar to the negative controls. Our results show that γ-UDL has an antifungal effect against *C. albicans* at the MFC, without hemodialysis as the observed cytotoxicity.

## Figures and Tables

**Table 1:** MIC and MFC of each chemical against *Candida albicans. n* = 3, mean ± SD.

Chemical	MCFA1	MCFA2	MCFA3	MCFA4	γ-UDL	δ-UDL	HFDEC
MIC (µg/mL)	225	225	112.5	112.5	112.5	>900	>900
MFC (µg/mL)	450	450	225	225	225	>900	>900

**Table 2:** Physicochemical properties of each chemical. (*: *p* < 0.05, multiple regression analysis).

Chemical		MCFA1	MCFA2	MCFA3	MCFA4	γ-UDL	δ-UDL	HFDEC
MW		144.2	158.2	172.3	186.3	184.3	184.3	492.1
pKa		5.19	5.23	4.95	4.95	No ionizable atoms found	No ionizable atoms found	0.11
Ion form fraction (pH 6.2)		91.20	91.20	95.50	95.50	0.00	0.00	100.00
Molecular form fraction (pH 6.2)		8.80	8.80	4.50	4.50	100.00	100.00	0.00
log *P*		2.44	2.84	3.23	3.63	2.87	2.87	6.77
log *D* (pH 6.0)		1.83	2.31	2.51	2.95	3.31	3.31	2.38
log *S* (pH 6.0)		−1.61	−2.18	−2.46	−2.99	−3.27	−3.04	−2.82
Polarizability *	isotopic	16.96	19.05	21.16	23.23	22.53	22.47	24.17
	tensor component xx	14.26	15.77	16.48	18.76	19.7	20.58	20.55
	tensor component yy	12.48	15.02	17.25	18.01	17.5	17.56	20.63
	tensor component zz	23.14	26.37	29.74	33.03	30.38	29.76	29.31

## Data Availability

The original contributions presented in this study are included in the article. Further inquiries can be directed to the corresponding author.

## References

[1] Desbois A., Smith V. (2010). Antibacterial free fatty acids: Activities, mechanisms of action and biotechnological potential. Appl. Microbiol. Biotechnol..

[2] Kabara J.J., Swieczkowski D.M., Conley A.J., Truant J.P. (1972). Fatty acids and derivatives as antimicrobial agents. Antimicrob. Agents Chemother..

[3] Venkata M.S., Rohit M., Chiranjeevi P., Chandra R., Navaneeth B. (2015). Heterotrophic microalgae cultivation to synergize biodiesel production with waste remediation: Progress and perspectives. Bioresour. Technol..

[4] Desbois A. (2012). Potential applications of antimicrobial fatty acids in medicine, agriculture and other industries. Recent Pat. Anti-Infect. Drug Discov..

[5] Centers for Disease Control and Prevention (CDC) (2020). Antibiotic/Antimicrobial Resistance. https://www.cdc.gov/drugresistance/.

[6] Yin H., Li G., Chen X., Wang W., Wong P.K., Zhao H., An T. (2020). Accelerated evolution of bacterial antibiotic resistance through early emerged stress responses driven by photocatalytic oxidation. Appl. Catal. B.

[7] Yoon B., Jackman J., Valle-Gonz’alez E., Cho N. (2018). Antibacterial free fatty acids and monoglycerides: Biological activities, experimental testing, and therapeutic applications. Int. J. Mol. Sci..

[8] Kumar P., Lee J., Beyenal H., Lee J. (2020). Fatty acids as antibiofilm and antivirulence agents. Trends Microbiol..

[9] Sanabria-Ríos D.J., Morales-Guzmán C., Mooney J., Medina S., Pereles-De-León T., Rivera-Román A., Ocasio-Malavé C., Díaz D., Chorna N., Carballeira N.M. (2020). Antibacterial activity of hexadecynoic acid isomers toward clinical isolates of multidrug-resistant Staphylococcus aureus. Lipids.

[10] Selvadoss P.P., Nellore J., Ravindrran M.B., Sekar U., Tippabathani J. (2018). Enhancement of antimicrobial activity by liposomal oleic acid-loaded antibiotics for the treatment of multidrug-resistant Pseudomonas aeruginosa. Artif. Cells Nanomed. Biotechnol..

[11] Isaacs C.E., Litov R.E., Thormar H. (1995). Antimicrobial activity of lipids added to human milk, infant formula, and bovine milk. Nutr. Biochem..

[12] Kabara J.J., Kabara J.J. (1978). Fatty acids and derivatives as antimicrobial agents. The Pharmacological Effect of Lipids.

[13] Clitherow K., BInaljadm T., Hansen J., Spain S., Hatton P., Murdoch C. (2020). Medium-Chain Fatty Acids Released from Polymeric Electrospun Patches Inhibit *Candida albicans* Growth and Reduce the Biofilm Viability. ACS Biomater. Sci. Eng..

[14] Thormar H., Isaacs C.E., Brown H.R., Barshatzky M.R., Pessolano T. (1987). Inactivation of enveloped viruses and killing of cells by fatty acids and monoglycerides. Antimicrob. Agents Chemother..

[15] Bergsson G., Arnfinnsson J., Steingrimsson O., Thormar H. (2001). In Vitro Killing og *Candida albicans* by Fatty Acids and Monoglycerides. Antimicrob. Agents Chemother..

[16] Kristmundsdóttir T., Árnadóttir S., Bergsson G., Thormar H. (1999). Development and evaluation of microbicidal hydrogels containing monoglyceride as the active ingredient. J. Pharm. Sci..

[17] Bergsson G., Steingrímsson Ó., Thormar H. (1999). In vitro susceptibilities of Neisseria gonorrhoeae to fatty acids and monoglycerides. Antimicrob. Agents Chemother..

[18] Bergsson G., Arnfinnsson J., Karlsson S.M., Steingrímsson Ó., Thormar H. (1998). In vitro inactivation of Chlamydia trachomatis by fatty acids and monoglycerides. Antimicrob. Agents Chemother..

[19] Isaacs C.E., Kim K.S., Thormar H. (1994). Inactivation of enveloped viruses in human bodily fluids by purified lipids. Ann. N. Y. Acad. Sci..

[20] Williams A.A., Sugandhi E.W., Macri R.V., Falkinham J.O., Gandour R.D. (2007). Antimicrobial activity of long-chain, water-soluble, dendritic tricarboxylato amphiphiles. J. Antimicrob. Chemother..

[21] McCrory C., Verma J., Tucey T.M., Turner R., Weerasinghe H., Beilharz T.H., Trave A. (2023). The short-chain fatty acid crotonate reduces invasive growth and immune escape of Candida albicans by regulating hyphal gene expression. mBio.

[22] Delmotte P., Delmotte-Plaque J. (1953). A new antifungal substance of fungal origin. Nature.

[23] Zhao A., Lee S.H., Mojena M., Jenkins R.G., Patrick D.R., Huber H.E., Goetz M.A., Hensens O.D., Zink D.L., Vilella D. (1999). Resorcylic acid lactones: Naturally occurring potent and selective inhibitors of MEK. J. Antibiot..

[24] Brautaset T., Sletta H., Degnes K.F., Sekurova O.N., Bakke I., Volokhan O., Andreassen T., Ellingsen T.E., Zotchev S.B. (2011). New nystatin-related antifungal polyene macrolides with modified polyol domains generated by biosynthetic engineering of Streptomyces noursei. Appl. Environ. Microbiol..

[25] Akinosoglou K., Rigopoulos E.A., Papageorgiou D., Schinas G., Polyzou E., Dimopoulou E., Gogos C., Dimopoulos G. (2024). Amphotericin B in the Era of New Antifungals: Where Will It Stand?. J. Fungi.

[26] Van Nguyen M., Han J.W., Kim H., Choi G.J. (2022). Curvicollide D, a new modified γ-lactone from the culture broth of Albifimbria verrucaria and its antifungal activity against plantpathogenic fungi. J. Antibiot..

[27] Sautour M., Lemaître J., Ranjard L., Truntzer C., Basmaciyan L., Depret G., Hartmann A., Dalle F. (2021). Detection and survival of *Candida albicans* in soils. Environ. DNA.

[28] Chaieb K., Kouidhi B., Zmantar T., Mahdouani K., Bakhrouf A. (2011). Starvation survival of *Candida albicans* in various water microcosms. J. Basic Microbiol..

[29] Whiteway M., Bachewich C. (2007). Morphogenesis in *Candida albicans*. Annu. Rev. Microbiol..

[30] Lee Y., Puumala E., Robbins N., Cowen L.E. (2021). Antifungal drug resistance: Molecular mechanisms in *Candida albicans* and beyond. Chem. Rev..

[31] Butassi E., Svetaz L., Carpinella M.C., Efferth T., Zacchino S. (2021). Fungal biofilms as a valuable target for the discovery of natural products that cope with the resistance of medically important fungi-latest findings. Antibiotics.

[32] Calderone R.A., Fonzi W.A. (2001). Virulence factors of *Candida albicans*. Trends Microbiol..

[33] Slobodnikova L., Fialova S., Rendekova K., Kovac J., Mucaji P. (2016). Antibiofilm activity of plant polyphenols. Molecules.

[34] Mulat M., Pandita A., Khan F. (2019). Medicinal plant compounds for combating the multidrug resistant pathogenic bacteria: A review. Curr. Pharmaceut. Biotechnol..

[35] Mori T., Yoshida M., Hazekawa M., Ishibashi D., Hatanaka Y., Nagao T., Kakehashi R., Kojima H., Uno R., Ozeki M. (2021). Antimicrobial activities of LL-37 fragment mutant-poly (lactic-co-glycolic) acid conjugate against *Staphylococcus aureus*, *Escherichia coli*, and *Candida albicans*. Int. J. Mol. Sci..

[36] Mori T., Yoshida M., Hazekawa M., Ishibashi D., Hatanaka Y., Kakehashi R., Nakagawa M., Nagao T., Yoshii M., Kojima H. (2021). Targeted Delivery of Miconazole Employing LL37 Fragment Mutant Peptide CKR12-Poly (Lactic-Co-Glycolic) Acid Polymeric Micelles. Int. J. Mol. Sci..

[37] Yoshida M., Hayashi S., Haraguchi T., Ito M., Hatanaka Y., Yoshii M., Tatsuoka H., Tanaka S., Nagao T. (2024). Antimicrobial activity of positively charged oligopeptides with theoretical high α-helix content against Cutibacterium acnes. Int. J. Mol. Sci..

[38] Haraguchi T., Hayashi S., Nakasaka S., Hatanaka Y., Nagao T., Tanaka S., Yoshii M., Hagimori M., Yoshida M. (2024). Antimicrobial activity of 2-(Piperazin-1-yl)naphtho [2,3-d]thiazole-4,9-dione against Staphylococcus strains. Molecules.

[39] Kannan T.R., Baseman J.B. (2000). Hemolytic and Hemoxidative Activities in Mycoplasma penetrans. Infect. Immun..

[40] Derkacz D., Krasowska A. (2023). Alterations in the Level of Ergosterol in Candida albicans’ Plasma Membrane Correspond with Changes in Virulence and Result in Triggering Diversed Inflammatory Response. Int. J. Mol. Sci..

[41] Antrey P.P., Hasen K.C. (1988). Role of Yeast Cell Growth Temperature on Candida albicans Virulence in Mice. Infect. Immun..

[42] Sanglard D., Ischer F., Parkinson T., Falconer D., Bille J. (2003). Candida albicans Mutations in the Ergosterol Biosynthetic Pathway and Resistance to Several Antifungal Agents. Antimicrob. Agents Chemother..

[43] Shiradhone A.B., Ingle S.S., Zore A.G.B. (2018). Microenvironment Responsive Modulations in the Fatty Acid Content, Cell Surface Hydrophobicity, and Adhesion of Candida albicans Cells. J. Fungi.

